# Proliferation of epithelial rests of Malassez following auto-transplantation of third molars: a case report

**DOI:** 10.1186/1752-1947-4-328

**Published:** 2010-10-19

**Authors:** Tom Struys, Joke Schuermans, Livia Corpas, Constantinus Politis, Luc Vrielinck, Serge Schepers, Reinilde Jacobs, Lambrichts Ivo

**Affiliations:** 1 Biomedical Research Institute, Hasselt University, Campus Diepenbeek, Diepenbeek, Belgium; 2 Department of Maxillo-Facial Surgery, Hospital of East Limburg (ZOL), Genk, Belgium; 3 Oral Imaging Center, Catholic University Leuven, Leuven, Belgium; 4 Department of Maxillo-Facial Surgery, Gent University, Gent, Belgium

## Abstract

**Introduction:**

Auto-transplantation of third molars is frequently undertaken in order to restore a perfect occlusion and to improve mastication following a substantial loss of molars. However, little is known about the precise role of the periodontal membrane during this procedure. Therefore, we investigated if the epithelial rests of Malassez persist in the periodontal ligament of auto-transplanted teeth and, if so, whether these may show signs of a neuro-epithelial relationship.

**Case presentation:**

We report a case of a 21-year-old Caucasian woman who underwent an auto-transplantation of two third molars. After two years, renewed progressive caries of the auto-transplanted teeth led to the removal of the auto-transplanted elements. The periodontal ligament was removed and studied with a light and transmission electron microscope.

**Conclusion:**

In this report we examined the ultrastructure of the periodontal ligament after auto-transplantation in order to see if the periodontal ligament recovers completely from this intervention. We observed fully developed blood vessels and a re-innervation of the epithelial rests of Malassez which were proliferating following auto-transplantation. This proliferation might be critical in the remodelling of the alveolar socket in order to provide a perfect fit for the transplanted tooth. In order to minimalise the damage to the epithelial rests of Malassez, the extraction of the tooth should be as atraumatic as possible in order to provide an optimal conservation of the periodontal ligament which will be beneficial to the healing-process.

## Introduction

The periodontal ligament (PDL) is the dense fibrous connective tissue which connects the cementum-covered surface of the root with the alveolar bone [1]. Its main function lies in preventing damage to the dental tissues during mastication. It consists, in part, of thick collagen bundles, called Sharpey's fibers, that run from the alveolar wall into the cementum and are responsible for resisting the displacing masticatory forces. Other functions which are addressed to the cells in the PDL are the formation, maintenance and repair of the alveolar bone and cementum. It has already been described that the alveolar bone can adapt its shape according to the needs during root development [2]. This is an important feature when looking at auto-transplantation where this process will be responsible for remodelling the new alveolar socket to the shape of the transplanted tooth. Furthermore, the periodontal ligament has rich sensory innervations [3] and a close relationship with the mechanoreceptors and the epithelial rests of Malassez (ERM) has been detected [4]. ERM are the remnants of the epithelial root sheath of Hertwig (ERSH), a fold of the outer and inner enamel epithelium formed during tooth development. Once root formation is completed, the ERSH becomes penetrated by several collagen bundles of the PDL, resulting in a fenestrated network that surrounds the tooth. The precise function of the ERM is not known yet, but it is believed that they are involved in preventing root resorption and maintaining the width of the periodontal ligament, thereby preventing ankylosis [5]. As it is reported that proliferation of ERM occurs during experimental tooth movement [6], the aim of this study was to investigate whether an auto-transplantation could also act as a trigger for this epithelial proliferation.

## Case presentation

A 21-year-old Caucasian woman presented to our clinic with multiple caries and inflammatory paradental cysts (IPCs). One of the IPCs was located in the lower jaw near molar 37. A histopathological examination revealed that the cyst was predominantly surrounded by granulation tissue although the local presence of Malpighian epithelium could be found. On the periphery it was surrounded by an inflammatory infiltrate which consisted mainly of lymphocytes, plasmocytes and neutrophilic polymorphonuclear cells. The outermost lining consisted of a dense compact connective tissue and no signs of malignant degeneration could be detected.

**Figure 1:**
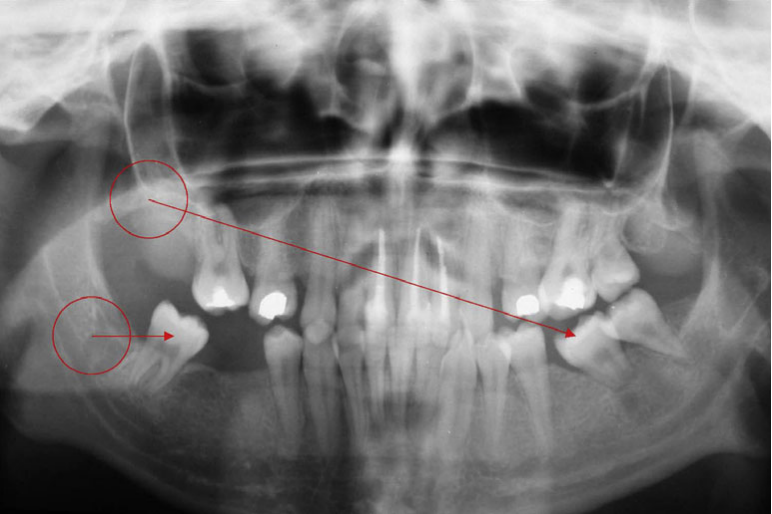
**X-ray of the upper and lower jaw four months after surgery**. Teeth 18 and 48 were extracted carefully and transplanted into position 36 and 47, respectively (marked by circles and arrows).

Two weeks later, the IPCs were enucleated after incision and trepanation of the bone. We decided to extract teeth 15, 37, 45 and 47 because of multiple and severe carious lesions. As the patient had a substantial loss of molars, the intra-osseous teeth 18 and 48 were extracted carefully and transplanted into position 36 and 47, respectively. The procedure was done as atraumatically as possible with no visible damage to the periodontal ligament of the extracted teeth. No problems were encountered during surgery and the auto-transplantation was a success. After four months, an X-ray was taken of the upper and lower jaw (Figure 1) with a Siemens Orthoceph 10E operated at 70 kV and 15 s of irradiation.

Two years later, the patient requested a partial extraction of the lower jaw teeth because of recurrent infections. As a result of renewed progressive caries of the two auto-transplanted teeth, she agreed with the removal of the auto-transplanted elements. The teeth were collected with her informed consent and the approval of the ethical board.

The extracted auto-transplanted teeth were immediately immersed and conserved in formol. The tissue of interest was collected by removing the PDL from the mid-cervical part of the teeth and it was fixed a second time in 2% glutaraldehyde in 0.05 M cacodylate buffer (pH 7.3). The fixative was gently aspirated with a glass pipette and the specimens were post-fixed in 2% osmium tetroxide, put through a dehydrating series of graded concentrations of acetone and embedded in araldite according to the conventional method. Semi-thin sections (0.5 μm) were stained with a solution of thionin and methylene blue (0.1 aqueous solution) for light microscopy. Ultra-thin sections (0.06μm) were mounted on 0.7% formvar-coated grids, stained with uranyl acetate and lead citrate and examined in a Philips EM 208 transmission electron microscope operated at 80 kV.

**Figure 2:**
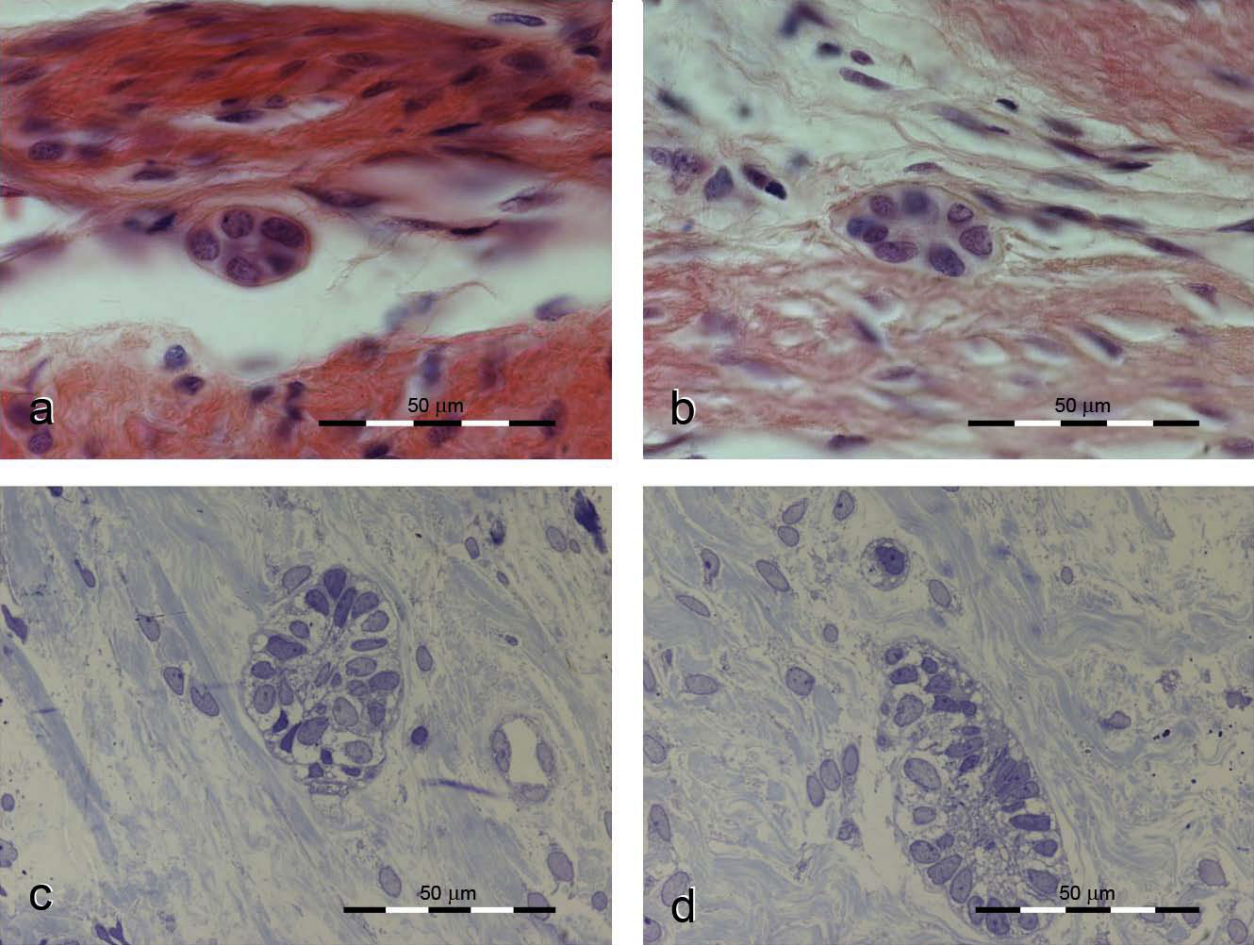
**Light microscopic images of epithelial rests of Malassez (ERM)**. (a-b) ERM of a normal human periodontal ligament with an average of 10 cells to 1 ERM. (c-d) ERM of a transplanted human periodontal ligament with an average of 20 cells to 1 ERM.

**Figure 3:**
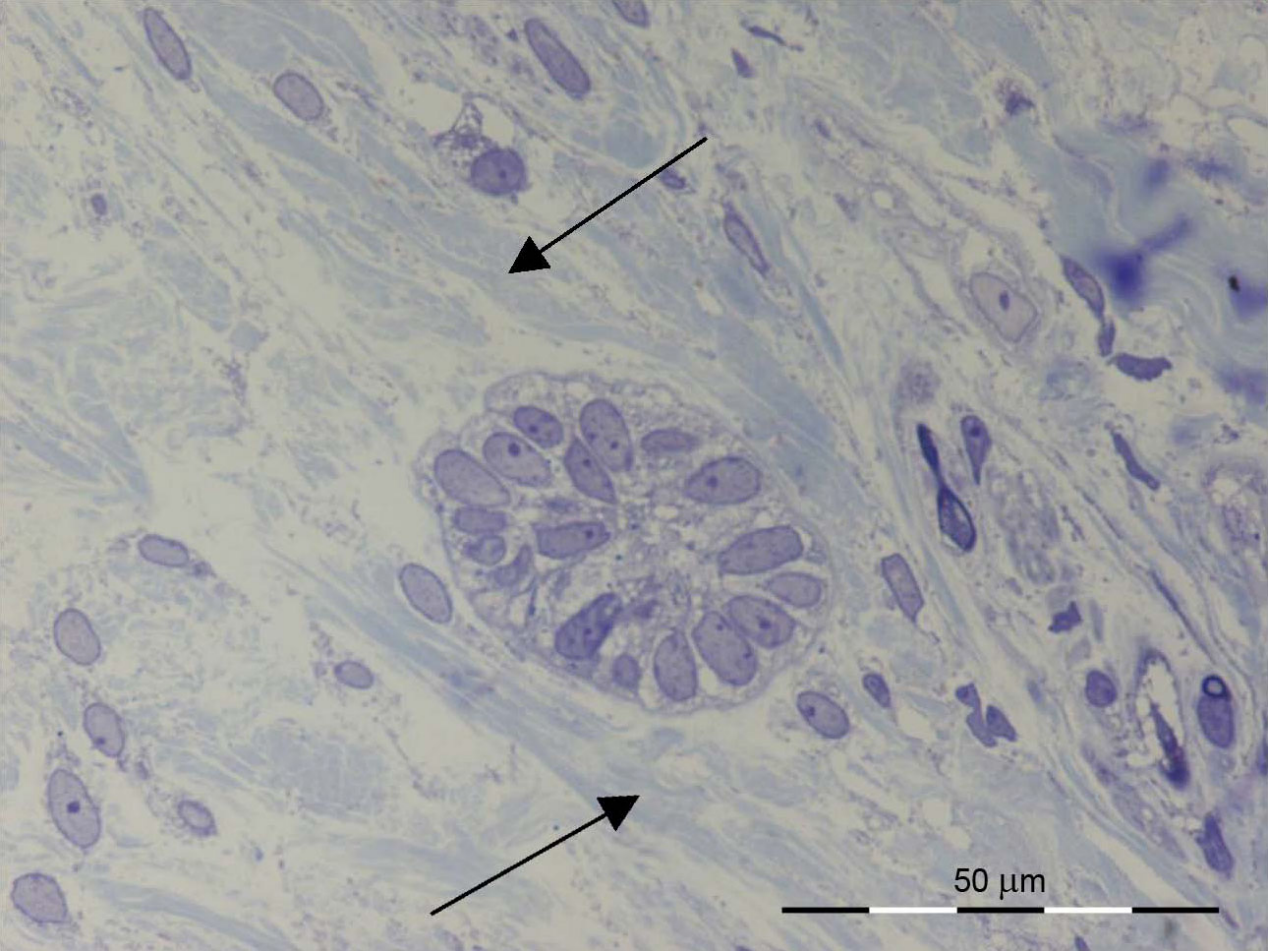
**Light microscopic image of the compartmentalization of collagen fibers that occurs after autotransplantation (arrow)**.

From a light microscopic examination of the semi-thin sections, we concluded that the ERM of the transplanted teeth were slightly larger than in normal PDL. A mean value of 20 cells was counted in the transplanted tissue in contrast to a mean value of 10 cells in normal/control PDL (Figure 2). We also noted compartmentalization of collagen bundles in the PDL (arrows in Figure 3).

**Figure 4:**
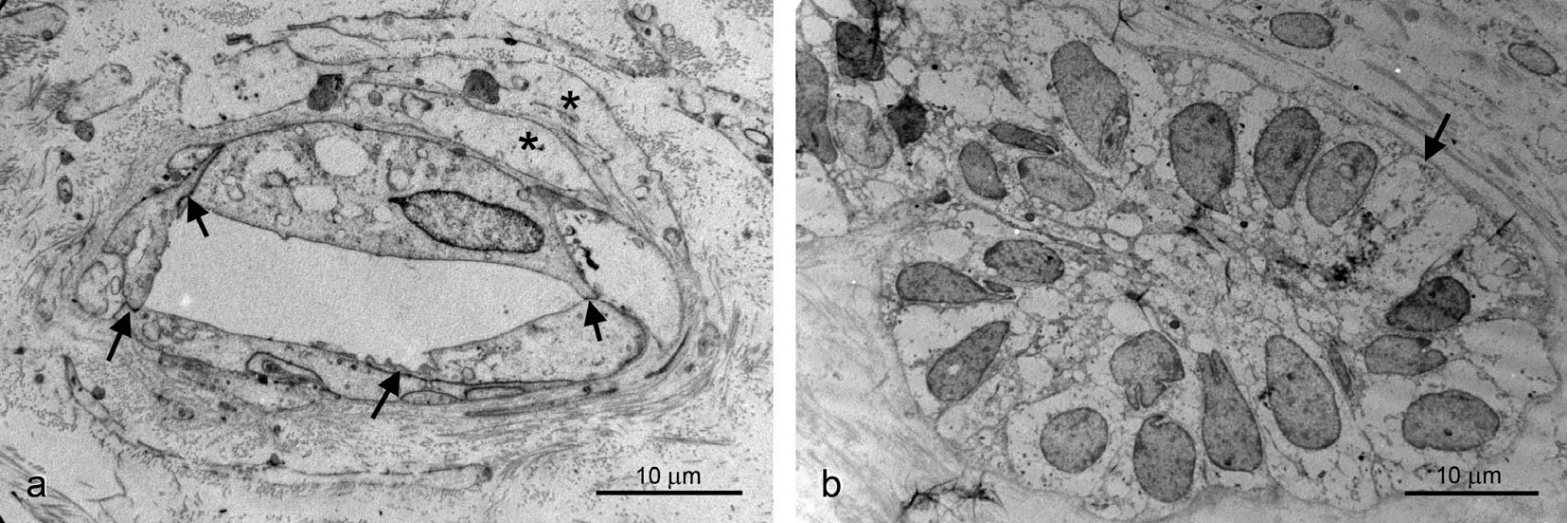
**Electron microscopic images of the transplanted PDL**. (a) Shows fully developed blood vessels. (b) Shows the electron microscopic aspect of the enlarged ERM.

**Figure 5:**
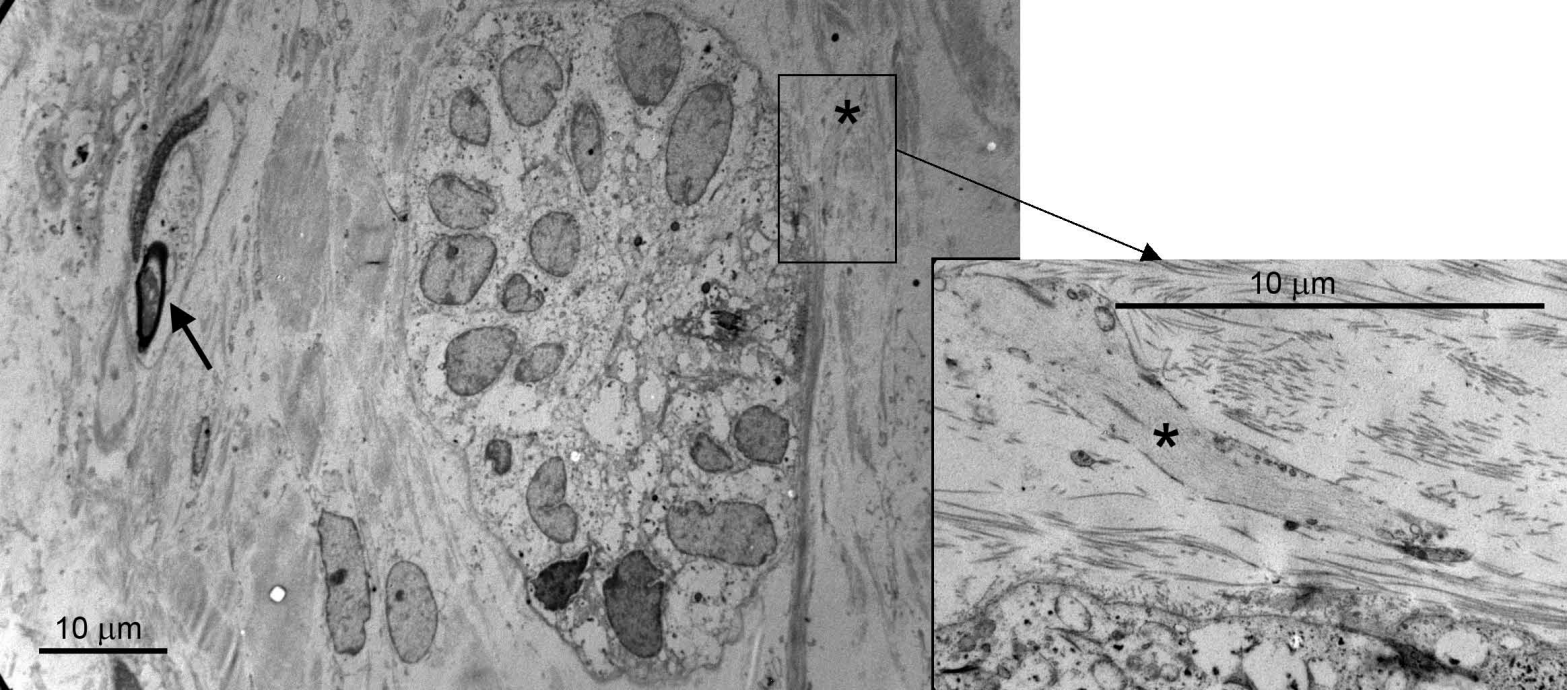
**Electron microscopic image of the re-innervation of the ERM in the autotransplanted PDL**.

From transmission electron microscope (TEM) analysis we concluded that the auto-transplantation was successful because fully developed blood vessels appeared in the PDL (Figure 4a). The lumen was surrounded by mature endothelial cells which were firmly connected to each other with tight junctions (arrows in Figure 4a). In the periphery, the blood vessels were supported by smooth muscle cells (asterisks in Figure 4a). The enlargement of the ERM seen with the light microscope was confirmed by the TEM images (Figure 4b). The epithelial cells formed typical clusters which were separated by bundles of collagen fibres. The epithelial nuclei were large, predominantly euchromatic and irregular in shape. The ERM were lined by a basal lamina (arrow in Figure 4b). Another interesting feature was the innervation of the ERM. Some fine neurites made contact with the ERM (Figure 5). These were characterized by the presence of neurofilaments in the cytoplasm (asterisks in Figure 5). Apart from these neurites, fully matured myelinated nerve fibres (arrow in Figure 5) accompanied by their Schwann cells were another feature of the successful regeneration of the PDL.

## Discussion

The specific morphological features which could be detected on the ultrastructural level can be regarded as typical for ERM and are confirmed in the recent literature [7-9]. ERM cells produce prostaglandin E2 [10] and prostaglandin E2 is capable of activating osteoclasts which stimulate bone breakdown and bone remodelling [11].

In auto-transplantation, the alveolar bone around the implantation-site normally has to be remodeled to provide a good fit for the implanted tooth. Bone breakdown is a process involved in this remodeling and it can be stimulated by increased prostaglandin E2 secretion by the ERM. This could explain why the ERM in the PDL of transplanted teeth are enlarged. It is also possible that the ERM in PDL of transplanted teeth remain enlarged when the remodeling process has finished. This implies that transplanted teeth will always have more mobility in the jaw than normal teeth because of the increased prostaglandin E2 secretion.

In addition to the expected bone remodeling, the PDL also needs to be remodeled. The compartmentalization of the collagen bundles can be seen as a consequence of this process. Furthermore, following auto-transplantation, the need for re-innervation of the PDL is of significant value. As ERM play an important role in the distribution of the fibrous and neural elements in the PDL[12], the enlargement of ERM detected after auto-transplantation could be seen as an attempt to direct PDL remodeling and re-innervation. The innervation of the ERM suggests that this whole process is directed by the nervous system.

## Conclusion

One of the key elements necessary for a successful auto-transplantation is the conservation of the periodontal ligament. These results suggest that the extraction should be as atraumatic as possible in order to conserve the periodontal ligament and minimalise the damage brought to the ERM.

## Abbreviations

ERM: epithelial rests of Malassez; ERSH, epithelial root sheath of Hertwig; IPC, inflammatory paradental cyst; PDL, peridontal ligament; TEM, transmission electron microscope.

## Competing interests

The authors declare that they have no competing interests.

## Authors' contributions

SJ, PC, SS and VL performed the surgical procedures, collected, analyzed and interpreted the patient data in order to determine the surgical follow-up. ST and LI performed the histological and ultrastructural examination of the PDL and wrote the manuscript. CL and JR revised the manuscript and contributed to the overall discussion. All authors read and approved the final manuscript.

## Consent

Written informed consent was obtained from the patient for publication of this case report and accompanying images. A copy of the written consent is available for review by the Editor-in-Chief of this journal.
